# Evaluating the potential applications of brewers' spent grain in biogas generation, food and biotechnology industry: A review

**DOI:** 10.1016/j.heliyon.2022.e11140

**Published:** 2022-10-19

**Authors:** Jovine K. Emmanuel, Philimon D. Nganyira, Godlisten N. Shao

**Affiliations:** University of Dar Es Salaam, Mkwawa University College of Education, Department of Chemistry, P.O. Box 2513, Iringa, Tanzania

**Keywords:** Brewers' spent grain, Anaerobic digestion, Biogas production, Cellulose and non-cellulosic polysaccharides

## Abstract

Breweries, as the major users of fossil fuels, are constantly under economic and environmental pressure to minimize energy consumption and residual management costs. Biogas generation from brewing wastes is a realistic solution for significantly reducing fossil fuel use. Brewers' spent grain (BSG) forms about eighty per cent of the total wastes from a brewing plant. BSG has a high cellulose and non-cellulosic polysaccharides content which makes it potential for biogas production. This paper reviews the potential applications of BSG as an alternative substrate for production of biogas and the recent achievements which have been attained in anaerobic digestion (AD) technology. The usability of BSG in diverse technologies including production of animal and human food and as a medium for growing microorganisms and enzymes is reviewed. The chemical processes involved in producing biogas from BSG are discussed.

## 1 Introduction

The residuals produced from the brewing industry are increasingly becoming more challenging and expensive to treat [1]. Per each 1000 tonnes brewing product, 137–173 tonnes of residuals (spent grain, wort trub and waste yeast) are created [2, 3]. Brewing by-products can be turned into a variety of economically viable and marketable goods which would significantly save the disposal costs and prevent environmental pollution [4]. Proper utilization of brewer’s spent grain (BSG) can support plant growth and improve human and animal nutrition [3, 5, 6]. BSG is the major waste generated from brewers which contains high protein. BSG is used as a supplement in human nutrition [4] and food for monogastric and ruminant animals [7, 8, 9]. There have been a number of attempts to employ BSG in biotechnological operations such as mushroom and actinobacteria culture [10, 11]. Revealing that BSG has a wide potential for many applications. Since BSG is released in a large amount, the discovery of novel strategies to utilize such brewery waste is vital [1, 7].

Specifically, BSG chiefly contains barley malt grain husks and a portion of the pericarp plus seed coat layers [10, 12, 13]. Although BSG contains sugars, proteins and minerals, the components are diverse and largely depend on the kind of barley used, harvest period and farming environments, malting and crushing settings and the amount and kind of additions [10, 14]. Table 1 lists BSG components as reported by different authors.

**Table 1:** BSG' components.

Components (dry matter) (g/100 g)	Khidzir *et al.* 2010 [15]	Waters *et al.* 2012 [16]	Meneses *et al.* 2013 [17]	Bianco *et al.* 2020 [18]	Marcus & Fox, 2021 [19]
Cellulose (glucan)	-	26.0	21.73 ± 1.36∗	0.3–33	21.9
Hemicellulose	-	22.2	19.27 ± 1.18∗	19.2–41.9	29.6
Xylan	-	-	13.63 ± 0.82	13.6–20.6	-
Arabinan	-	-	5.64 ± 0.35	5.6–41.9	-
Starch	-	-	-	1.0–12.0	13.0
Lignin	-	-	19.40 ± 0.34	11.5–27.8	22.0
Lipids	2.3 ± 0.8	-	-	3.0–10.6	10.6
Proteins	6.4 ± 0.3	22.1	-	14.2–31.0	26.7
Ashes	-	1.1	4.18 ± 0.03	1.1–4.6	4.6
Extractives	2.3 ± 0.8	-	-	5.8–10.7	-
Phenolics	-	-	-	0.7–2.0	2.0
Insoluble in acid	-	-	17.54 ± 0.31	-	-
Soluble in acid	-	-	1.86 ± 0.03	-	-

The management of BSG is one of the challenges that all brewers face [20]. The study by Ferreira et al. [20] reported the most frequent ways for managing BSG include; producing animal fodders, supplying it to nearby farmers, and dumping it to a landfill. The last is less attractive due to expenditures incurred by brewers in addition to the environmental concerns. Various investigations (Ferreira et al. and the reference therein) [20] have shown that BSG has a huge potential and can be used as a source of food, a medium for culturing microorganisms and production of enzymes and biogas generation. Reports address the importance of developing valuable BSG-based goods through extracting the unused components, particularly in terms of food security, economics and environmental sustainability [21]. The present review discusses the potential applications of BSG to provide a wide understanding on how this material can be effectively utilized to avoid the disposal cost and prevent environmental pollution. The generation of biogas from BSG is described herein.

## 2 Overview on the applications of brewery industry wastes

BSG is an important starting substrate because it is affordable, accessible and its components have high value [10]. Initiatives to incorporate BSG into food, biogas generation, and technological routes are addressed.

### 2.1 Brewers' spent grain - a source of animal food

The most common usage of BSG is in animal feeding either wet or dry [10]. The large fiber and protein content and low cost make it the ideal material for this application. When compared to low-cost nitrogen precursors like urea, it provides the required amino acids for animal nutrition [10]. Furthermore, when BSG is included in cattle diets, it boosts milk production and total solids content while lowering fat level in the milk produced [10, 22]. The significant benefits of including BSG in cattle feeding prompted the use of this material to feed animals like poultry, pigs and fish [11].

### 2.2 Inclusion of brewers' spent grains in human nutrition

Brewers' spent grain has been deemed a valuable material for incorporation into human diets [10]. Its application in improving the nutritional value in bakery products [23] including breads, biscuits, cookies, muffins, cakes, waffles, pancakes, tortillas, snacks, doughnuts and brownies [24]. The health advantage of BSG are due to its large fiber content (arabinoxylans (AX), β-glucans) and phenolics (e.g. hydroxycinnamic acid) [25]. When BSG is used as an ingredient in bakery products and sandwiches, it improves the content of bioactive antioxidant and AX as well as a low glycemic index [26]. Since BSG is too granular in its native form, it should be transformed into flour before being used in extruded snacks. The incorporation of functional ingredients in baked goods lessens the danger of chronic diseases in addition to nutritional purpose [27]. However, only 15% BSG is thought to have a negative impact on sensory attributes [27]. According to McCarthy et al. [28], bread made with wheat flour and subjected to four enzymes and with BSG of up to 30% had a longer shelf life, enhanced texture and volume [28]. When 10% BSG is added to bread, the protein increases by 50%, fiber increases by 10%, and the crucial amino acid amount increases by 10% relative to bread that has no BSG [25, 29].

Additionally, BSG has a high content of bioactive phenolic molecules that have antioxidant properties and which can be extracted with a variety of approaches; solid–liquid isolation, microwave-assisted isolation, and enzymatic and alkaline reactions [10]. Of the mentioned approaches, solid–liquid isolation employing sixty percent (v/v) acetone/water solvent for 30 min at 60 °C was reported as the most effective approach for extracting antioxidant phenolic components from BSG [10].

Gmoser et al. [30] investigated the creation of nutrient-rich fungal-fermented materials from stale bread and BSG. The product was prepared by six days of solid state fermentation at 35 °C and 95% relative humidity and 40% moisture [30]. The use of fungal solid state fermentation to change stale bread and BSG into a nutrient-rich food product has been proven as an effective technique in reducing food wastes and protein shortages [30].

### 2.3 Brewers' spent grain – a support for micro-organisms growth and creation of enzymes

BSG is primarily applied to promote fungal development that produces enzymes (alpha-amylases, cellulases and hemicellulases) with amino acids, vitamins and inorganic molecules included to boost enzyme supply [31, 32]. Mussatto [33] reported that *Pleurotus*, *Agrocybe*, *Lentinus* and *Trichoderma* as well as *Streptomyces* bacteria thrive on BSG medium. BSG is used to isolate and maintain the appropriate strains for selection and synthesis of novel biologically active compounds [32, 34].

### 2.4 Brewers' spent grain - a substrate for energy generation

Thermochemical transformation (pyrolysis and combustion) and biogas and ethanol generation are alternatives for BSG utilization in energy production [10]. The current global energy demand has prompted the development of BSG based energy production [1, 10]. Therefore, the wide accessibility of BSG in addition to its components and affordability makes it an attractive starting substrate for this field, thus attracting global attention [20].

#### 2.4.1 Thermochemical conversion technology

The use of thermochemical transformation technologies like pyrolysis [35] and combustion [36] is among the available options for generating biogas from biomass substrates such as BSG. BSG has net and gross calorific values of 18.64 and 20.14 MJ kg^−1^ dry mass [10, 36], and is a promising starting substrate for heat generation through combustion [10]. In this process, BSG moisture content is reduced below 550 g kg^−1^ before combustion at high pressure [10]. The heat released by BSG combustion might meet the energy requirement of brewery industries [10]. However, BSG burning produces particles and hazardous gases containing nitrogen oxides and sulfur dioxide at concentrations between 1000–3000 and 480 mg m^−3^ [10, 36]. Thus, extra care is required when performing BSG combustion in order to avoid or reduce the side effects [36]. A further intriguing option is to use BSG to make charcoal bricks [10, 35, 37]. The manufacturing process begins with the drying of BSG accompanied by pressing and carbonization in a reduced-oxygen environment. The energy content of charcoal bricks generated by this technique is significant (27 MJ kg^−1^) and comparable to the energy content of charcoal manufactured from other raw resources such as wood, sugarcane, grape bagasse, olive bagasse, and hazelnut shell [10, 38, 39]. Transitional pyrolysis of BSG at 450 °C using a Pyroformer, a dual coaxial screw reactor produces 29% char, 51% bio-oil, and 19% perpetual vapours [10].

#### 2.4.2 Brewers' spent grain - a starting material for biogas generation

Because a large amount of BSG is generated per year, its affordability, nutritional content and increasing energy expenses make biogas generation from BSG a promising alternative that attracts global interest [10]. Reports suggest that BSG is used to produce biogas [10, 24, 40]. This application is particularly well suited to obtaining thermal energy in breweries with minimal environmental damage, and thus, BSG is a crucial substrate for biogas generation [41]. However, BSG contains a large amount of lignocellulosic substance [10] that degrades slowly under anaerobic circumstances [1]. Furthermore, biogas production is limited by phenolic intermediates of lignocellulose breakage like p-cresol [41]. In anaerobic co-digestion, a digestion of a combination of two or more different substrates is carried out aiming at enhancing the effectiveness of the process [42]. Anaerobic co-digestion has a number of advantages over single material digestion including; cost efficiency, biodegradability of the treated materials and greater biogas output [42]. The introduction of associated materials improves nutritional stability while lowering the amount of inhibitory intermediates, allowing the microbial communities to flourish thus, increasing biogas generation by 40–80% [1].

## 3 Biogas production

Biogas contains gases released by anaerobic consumption of organic matter by methanogenic bacteria, termed methane fermentation [43]. The process involves anaerobic methanogenic microbial actions from nearly any sort of organic waste [44]. Reports show that methane and carbon dioxide are the main components of dry biogas, but may include nitrogen, hydrogen, oxygen, hydrogen sulfide, ammonia and hydrocarbons [43]. Biogas may also contain traces of organic sulfur, chlorine, fluorine and silicon compounds along with aerosol emission [45]. However, biogas content is highly influenced by the substrate and process parameters [2, 12].

Biogas generation provides a variety of benefits including different fuel, quality fertilizer, energy, heat, total waste reuse, greenhouse gas minimization and avoids pollution [46]. Biogas is a flexible and dependable alternative to fossil fuels subsidising renewable and sustainable energy generation [46]. Biogas technology is used on a local or large scale in urban and rural settings [47]. Biogas has been produced by using a variety of feed stocks and reactor set-ups [47]. Table 2 presents the biogas content as reported in various studies.

**Table 2:** Biogas content [5, 48, 49].

Constituent	Formula	Percentage (%)
Methane	CH_4_	40–75
Carbon dioxide	CO_2_	25–60
Carbon monoxide	CO	0–0.1
Nitrogen	N_2_	0.5–2.5
Hydrogen	H_2_	1–3
Hydrogen sulfide	H_2_S	0.1–0.5
Oxygen	O_2_	0.1–1
Ammonia	NH_3_	0.1–0.5

### 3.1 Biogas production from a co-digestion of brewers' spent grain with various substrates

Mussatto et al. [12] studied BSG characteristics and the potential feasibility of utilizing BSG in energy generation by direct combustion or fermentation. The combustion process requires BSG to be dried to ≤55% moisture, and is complicated by the production of NOx and dust particles [12]. Anaerobic fermentation is a substitute for energy recovery from BSG but hydrolysis of the fibre in BSG is a restrictive step for conversion of the substrate into biogas [12].

Tewelde et al. [7] evaluated the generation of biogas from anaerobic single stage co-digestion of brewery wastes (BW) and cattle dung (CD). At mesophilic conditions, a mixture of BW and CD was evaluated in batch mode. A transformation of 73.8% of the organic materials in a digester was attained for each single stage batch digestion with retention duration of 40 days. This study reported an average gas yield of 0.290 m^3^ kg^−1^ VS^−1^ (Volatile Solids). The primary total solids content (TS) was altered from 16 to 8 percent by diluting with water in order to provide suitable fermentation conditions. The highest overall methane productivity was achieved at a CD: BW ratio of 70:30 and the highest organic filling rate of 3.3 kg VSm^−3^ d^−1^ produced from semi-continuous consumption at this 70:30 in the absence of the digester clogging. The details are presented in Table 3.

**Table 3:** Relationship between experimental data collected from various ratios of CD and BW with Hydraulic Retention Time (HRT) of 40 Days as reported by Tewelde et al. [7].

Ratio of feed content	TS%	Produced biogas, Lday^−1^	Biogas yield, m^3^kg^−1^VS^−1^_added_	Methane yield, m^3^CH_4_kg^−1^ VS^−1^_added_
CD	8	0.41	0.17	0.098
CD:BW 90:10	8	0.60	0.22	0.140
CD:BW 80:20	8	0.77	0.29	0.182
CD:BW 70:30	8	0.90	0.41	0.287
CD:BW 60:40	8	0.58	0.36	0.190
CD:BW 40:60	8	0.48	0.32	0.189
CD:BW 20:80	8	0.30	0.28	0.170

It was found that stable anaerobic co-digestion is obtained by employing a combination of brewery wastes and cattle dung in varied quantities. Inclusion of BW boosted the biogas generation from 0.4 LL^−1^ day^−1^ to 0.9 LL^−1^ day^−1^ [7].

Reports show that anaerobic digestion of thermal pretreated BSG increases biogas generation [50]. To improve anaerobic degradation, BSG is thermally pretreated which increases biogas generation [50]. BSG was pretreated at a temperature ranging between 100 and 200 °C [50]. The biogas generation from thermally pretreated BSG was between 30 and 40% greater than that of untreated BSG [50]. Biogas production increased at temperatures up to 160 °C. Temperatures over 160 °C led to slower degradation and decreased biogas yield [50].

Malakhova et al. [1] investigated the biotransformation of BSG in co-digestion with Jerusalem artichoke (JA, *Helianthus tuberosus L*.) phytomass through thermophilic (+55 °C) and mesophilic (+30 °C) anaerobic digestion into biogas. The nutrient medium included yeast extract and trace element solution to optimize the conditions for microbial growth [1]. BSG concentrations of 50 and 100 g L^−1^ were tested for biogas production [1]. When the inoculum was introduced, the total estimated methane production reached 64% under thermophilic conditions, consisting of roughly 6–8 and 9–11 L of CH_4_ per 100 g of fermented BSG in the absence and with co-digested JA [1]. The growth of lettuce (*Lepidium sativum* L.) was aided by the addition of residual fermented BSG (10 percent, w/w) to the soil [1]. Findings show the feasibility of complete utilization of BSG for biogas production and soil addition.

Evaluation of the influence of adding dried BSG on biogas generation and kinetics in co-digestion with sewage sludge (SS) is available [45]. Evaluation was carried out in semi-continuous anaerobic reactors (supplied once a day) working under mesophilic environments (35 °C) at various HRT of 18 and 20 days [42]. The BSG mass to feed volume ratio was kept at 1:10. In contrast to SS mono-digestion, the data show that adding BSG had no effect on biogas output (control run) [45]. For co-digestion experiment, the average methane output was 0.27 m^3^ kg^−1^ VS^−1^_added_ after an HRT of 18 days [45]. The greater value of 0.29 m^3^ kg^−1^ VS^−1^_added_ was observed in the control experiment (sewage sludge mono-digestion) [45]. For mono- and co-digestion experiments, the methane output was 0.21 m^3^ kg^−1^ VS^−1^_added_ at 20 days HRT [45]. Including of BSG, a drop in kinetic constant values was measured [41, 45]. In contrast to SS mono-digestion, a decline by 21 and 35% was noted at HRT of 20 and 18 days [45]. Supplementing the feedstock with BSG rich in organic compounds on the other hand resulted in a large increase in biogas production with the largest value of approximately 40% associated to the longer HRT of 20 days [41, 45]. More significantly, the mono- and co-digestion processes ran smoothly [45]. Szaja et al. [45] reported that anaerobic co-digestion of SS and BSG is seen as an economical option to help wastewater treatment plants (WWTPs) become more energy self-sufficient and breweries manage waste sustainably [41, 45]. Table 4 compiles the suitable substrates for biogas generation through BSG co-digestion.

**Table 4:** Biogas produced from brewers' spent grain co-digested with various substrates.

Substrates	Reference	Biogas produced	Optimum ratio used
Cow dung (CD) + Brewers' Spent Grain (BSG)	Tewelde *et al.* 2012 [7]	3.3 m^3^CH_4_kg^−1^VS^−1^d^−1^	70:30
Brewers' Spent Grain (BSG) + Jerusalem artichoke phytomass (JA)	Malakhova *et al.* 2015 [1]	0.06 m^3^ CH_4_Kg^−1^VS^−1^d^−1^	-
(Cow dung (CD) + Pig manure) + Brewers' Spent Grain (BSG)	Poulsen *et al.* 2017 [51]	85% of Ultimate CH_4_ yield	2:1
Brewers' Spent Grain (BSG) + Sewage Sludge	Szaja *et al.* 2020 [45]	0.27 m^3^ kg^−1^VS^−1^	1:10

From Table 4, it is noticed that the co-digestion of BSG with various substrates have enhanced the quality of the produced biogas. Co-digestion of brewers' spent grain with cow dung is highly recommended due to the easy availability of cow dung, pH of the cow dung, which is above 7, serves as a neutralizing agent to raise the pH of brewers' spent grain which is below 7.

## 4 Anaerobic digestion of biodegradable organic wastes

Anaerobic digestion is the decomposition of biodegradable substrate by microbes in a limited supply of oxygen [52]. It produces biogas that is high in methane and fertilizer that is high in nutrients while reducing the volume, bulk and toxicity of the input substrate [53]. Anaerobic digestion of continually created organic residues is a best alternative and an environmentally acceptable technique for reducing organic residues and environmental pollution while producing methane as an alternative energy [46, 54, 55]. The potentially beneficial lignocellulosic substrates include; energy crops, harvest residues, manure, home waste, wood industry waste and brewery by-products [56]. Lignocellulose is the world’s most plentiful renewable reserve which is readily available and inexpensive although its hydrolysis is frequently imperfect because of its complicated structure [8].

The rate-limiting phase in the AD of lignocellulosic substrate (LCS) that is used for biogas production is cellulose and hemicellulose solubilisation [8]. The breakdown of lignin is inefficient and produces hazardous intermediates upon digestion while crystalline cellulose is structurally very stable [41]. Thus, without further substrate treatment prior or in the course of biogas generation, biogas generation is typically inefficient [8, 46, 57].

The peculiarities of feedstock greatly affects the performance of anaerobic digestion [58]. The process of converting organic matter into biogas occurs in four stages: hydrolysis, acid formation, acetogenesis and methane production [4, 11]. Complex organic molecules are fragmented into amino acids, fatty acids, simple sugars and glucose during the hydrolysis stage [54]. Acidogenic bacteria transform the hydrolysis products into simple organic molecules, typically short-chain (volatile) acids in acidogenesis process [53]. Different groups of bacteria participate in different stages which are carried out simultaneously and constitute an anaerobic food chain in which products from one group become the substrate for another group [53]. The process goes on smoothly if the rates in several stages are balanced [4, 7, 11]. The degradation of feedstock in the limited supply of oxygen is enhanced by a mixture of microorganisms available at every step of the digestion resulting in the synthesis of digestate and a combination of gases including chiefly CH_4_ [53, 58].

## 5 Future prospects

Sustainable development goal number seven [59] highlights the importance of ensuring that everyone has access to affordable, dependable, sustainable, and modern energy. The current energy crisis and environmental demands facing developing countries are critical and call for exploration and proper utilization of alternative energy sources. The technology of biogas production has been used over the decades using varieties of organic wastes. The utilization of BSG is even more important in the current century where developing countries are embarking on the mission to reduce the consumption of fossil fuels.

The energy generated from BSG has been reported to exhibit higher calorific value compared to other organic wastes. However, its low degradability stimulated by the presence of high content of lignin and cellulose hampers its large-scale consumption. Notwithstanding, substantial investigations are being carried out to improve the production of biogas from this potential substrate. Therefore, to enhance the profitability of AD, biogas yield from BSG should be improved through different pretreatment approaches. Studies show that various pretreatment methods improve digestibility of lignin and cellulose contained in BSG which includes physical, thermal, chemical, biological and hybrid pretreatments [60, 61, 62, 63]. However, physical pretreatment is the foremost step of substrate preparation for better biogas yield [55, 60] which include; grinding, chipping, mechanical refining, cavitation, grubben deflaker and krima disperser, and Hollander beater [60] though is industrially expensive to implement [55, 61]. Thermal pretreatment method on the other hand include; hydrothermal-liquid hot water extraction, microwave heating, extrusion, torrefaction and steam explosion [62] although is not cost effective because of high energy input at high temperature and pressure [55, 60]. Besides, chemical pretreatment include; acidic pretreatment, alkaline pretreatment, redox reactions and Fenton reactions, and ionic liquid but is environmentally unfriendly [60]. Biological pretreatment is another approach which include; ensiling, fungi, micro-aeration, microbial consortium [60, 63, 64] and enzymatic pretreatment. Hybrid pretreatment is another approach which include wet oxidation and ammonia freeze explosion [65]. Of all pretreatment approaches for enhancing biogas yields, biological pretreatment is the more promising because it is cheap and environmentally friendly thus, is forecasted to replace the costly physico-chemical approaches [55, 60].

Interestingly, the combination of BSG with other substrates during AD produces a large quantity of biogas. Anaerobic co-digestion of BSG with CD is considered as the main focus of future research since the latter is very ubiquitous in majority of the developing countries. Table 5 summarizes the prospects of BSG and the process involved in making the material useful.

**Table 5:** Applications exploited from BSG and the process involved.

Reference	Application exploited from BSG	Process
Missana et al. (2022) [66]	Biogas production	Anaerobic digestion of brewery wastes.
Wagner et al. (2022) [67]	Bioethanol production	Fermentation of BSG using ethanologenic *Escherichia coli* MS04
Ofon et al. (2018) [24]	Biogas generation from co-digestion of BSG and palm oil mill effluent	Anaerobic co-digestion of palm oil mill effluent and BSG by incubating the reactors at 35 ± 2 °C for 30 days.
Panjičko et al. (2017) [44]	Biogas generation from BSG as a substrate	Two-phase anaerobic digestion (solid state anaerobic digestion reactor for hydrolysis and acidogenesis, granular biomass reactor for methanogenesis).
Fărcaș et al. (2021) [68]	Effect of BSG by-products on the nutritional contents, functional characteristics and sensorial profile of developed food products	Various kinds [5] of BSG were obtained from different local breweries and commercialized in cookies manufacturing.
Jackowski et al. (2021) [69]	Improvement of the fuel properties using torrefied BSG and using torrefied BSG as a beer colouring agent.	Torrefaction of BSG was conducted at temperatures ranging between 180 °C and 300 °C, with a residence time between 20 and 60 min, resulting in formation of a torrefied BSG with higher heating value of 25 MJ/kg.
Maqhuzu et al. (2021) [25]	Energy and food production	Hydrothermal carbonization involving artificial coalification of biomass by direct injection of steam in a pressurized vessel to biofuels with characteristics similar to coal and flour production
Neylon et al. (2021) [14]	Elevate techno-functional properties and nutritional value in high fibre bread	Fortification of wheat flour was carried out using spray-dried BSG and fermented BSG resulting to formation of dough with enhanced stiffness or resistance.
Amoriello et al. (2020) [23]	Bakery products enriched with BSG	Blending wheat flour with BSG flour at different levels (0%, 5% and 10%) to obtain new formulations for bakery products.
Gmoser et al. (2020) [30]	Using stale bread and BSG to produce protein-enriched products	Solid state fermentation using edible filamentous fungi *Neurospora intermedia* and *Rhizopus oryzae* at 35 °C, with 95% relative humidity and moisture content of 40% in the substrate for six days.
Szaja et al. (2020) [45]	Effect of BSG on biogas yields and kinetics	Anaerobic co-digestion of BSG with sewage slurry is carried out at 35 °C at different hydraulic retention.
Tan et al. (2020) [21]	Nutritional beverage production	Submerged fermentation of BSG using *Bacillus subtilis WX-17* without supplementary components. Fermentation products were extracted in the liquid phase, resulting in a potential novel nutritional beverage containing *Bacillus subtilis WX-17*
Ferreira et al. (2019) [20]	Production of combustible (H_2_ and CH_4_) gas from BSG	Steam gasification of BSG was conducted to discover the influence of steam to biomass ratio and temperature on the composition of the produced gas.
Zhang & Wang (2016) [35]	Production of biochar from BSG and sewage sludge for ammonia-nitrogen capture	A pyrolysis of BSG and sewage sludge (rich in magnesium and phosphorus) is carried out at 400–700 °C to yield biochars with potential use in removal of ammonia-nitrogen in aqueous solutions.
Zhang & Zang (2016) [70]	Biohydrogen production	Treatment of BSG samples with various concentrations (0.0–20 g/L) of calcined-red mud at 55 °C for 48 h to enhance fermentative hydrogen yield.

Reports show that the potential for BSG to be used for energy and food in Africa as well as its global warming potential imply that BSG has a lot of promise [25]. It is now well understood that BSG can be treated using hydrothermal carbonization technique to create an improved biofuel with properties similar to coal [25]. Wagner et al. [67] reported on bioethanol synthesis from BSG in a single pot using ethanologenic *Escherichia coli* MS04. The study revealed that bioethanol is cheaply obtained from BSG as well. Bioethanol is a very crucial bioenergy essential in transportation, chemical raw material or gasoline additive [67]. According to Amoriello et al. [23], BSG is considered as one of the most common and inexpensive brewing by-products since it has a variety of advantages in adding nutritional value to food products. Therefore BSG is used as a raw ingredient in bakery items because it is high in dietary fiber and protein [23]. These studies signify that BSG has several potentials and its production should not be considered as a calamity. However, further researches to explore the full potentials of BSG in food and energy technologies are indisputably fascinating and commendable.

## 6 Conclusions

The present review unveiled the potentials of brewing industry wastes a diverse technology as well as the future prospects. Studies have shown that the use of BSG in various technologies continues to attract global interest.

Despite the technological challenge facing most of the African countries, most studies show that the use of BSG in biogas technology, in food and biotechnology have the potential in providing breweries and the community with reliable and sustainable energy production, waste management, food security and the industrial cultivation of microorganisms and production of enzymes. Notwithstanding, the current review has uncovered the usability of BSG, a disregarded and environmentally stubborn brewery by-product, in human nutrition, animal food and biotechnology industry along with its future prospects. This provides the globe with a common understanding of the usability of BSG.

## Declarations

### Author contribution statement

All authors listed have significantly contributed to the development and the writing of this article.

### Funding statement

This research did not receive any specific grant from funding agencies in the public, commercial, or not-for-profit sectors.

### Data availability statement

No data was used for the research described in the article.

### Competing interest statement

The authors declare no conflict of interest.

### Additional information

No additional information is available for this paper.
